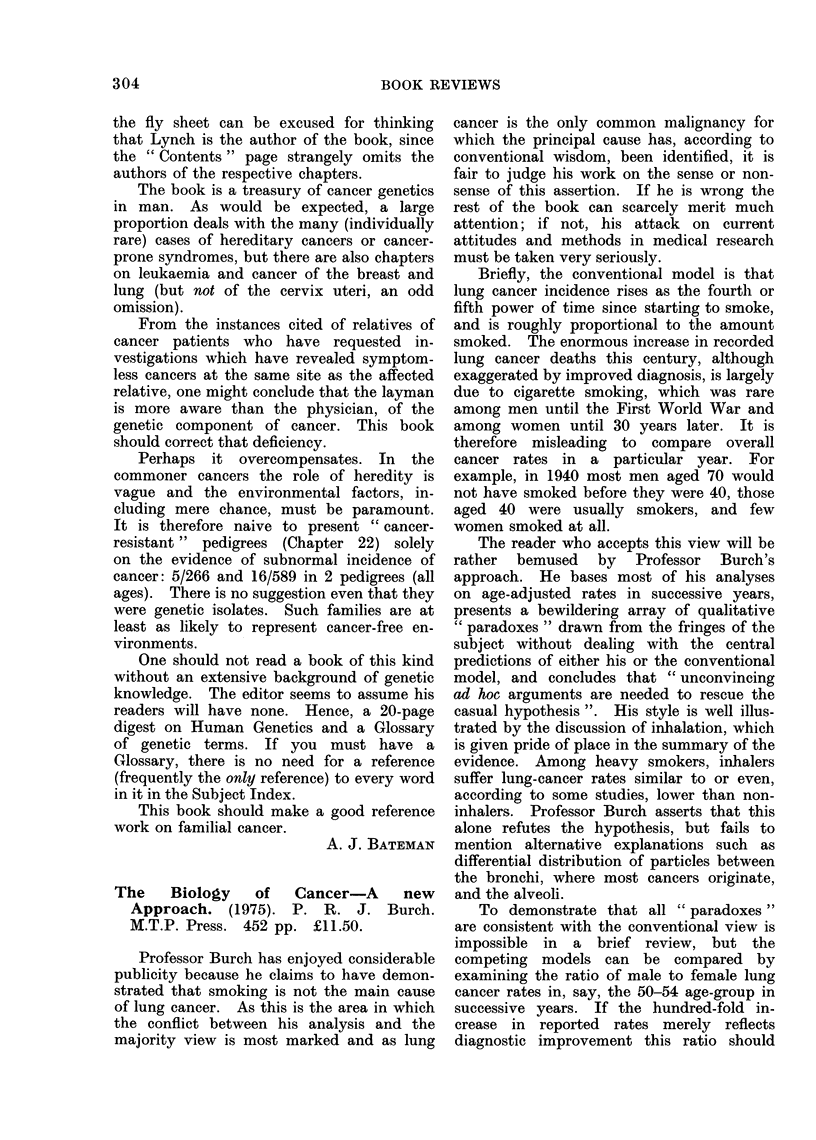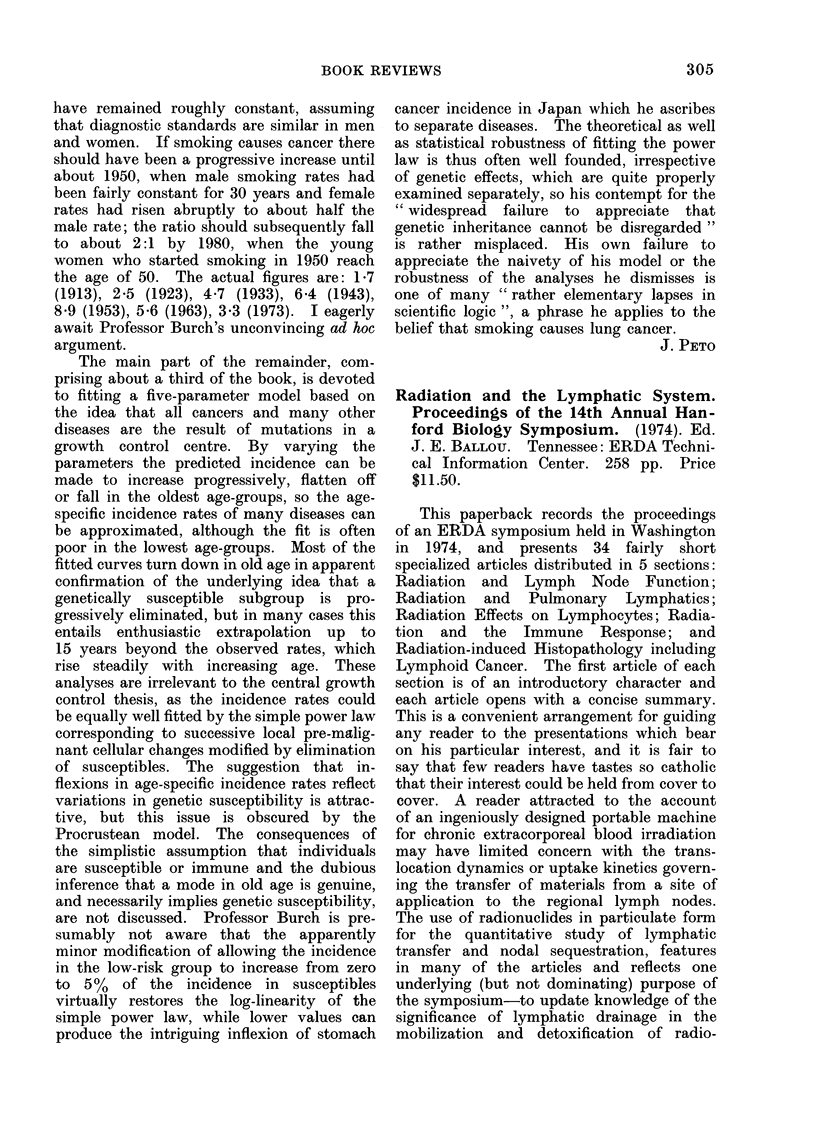# The Biology of Cancer—A new Approach

**Published:** 1976-09

**Authors:** J. Peto


**The Biology of Cancer—A new Approach.** (1975). P. R. J. Burch. M.T.P. Press. 452 pp. £11.50.

Professor Burch has enjoyed considerable publicity because he claims to have demonstrated that smoking is not the main cause of lung cancer. As this is the area in which the conflict between his analysis and the majority view is most marked and as lung cancer is the only common malignancy for which the principal cause has, according to conventional wisdom, been identified, it is fair to judge his work on the sense or nonsense of this assertion. If he is wrong the rest of the book can scarcely merit much attention; if not, his attack on current attitudes and methods in medical research must be taken very seriously.

Briefly, the conventional model is that lung cancer incidence rises as the fourth or fifth power of time since starting to smoke, and is roughly proportional to the amount smoked. The enormous increase in recorded lung cancer deaths this century, although exaggerated by improved diagnosis, is largely due to cigarette smoking, which was rare among men until the First World War and among women until 30 years later. It is therefore misleading to compare overall cancer rates in a particular year. For example, in 1940 most men aged 70 would not have smoked before they were 40, those aged 40 were usually smokers, and few women smoked at all.

The reader who accepts this view will be rather bemused by Professor Burch's approach. He bases most of his analyses on age-adjusted rates in successive years, presents a bewildering array of qualitative " paradoxes " drawn from the fringes of the subject without dealing with the central predictions of either his or the conventional model, and concludes that " unconvincing *ad hoc* arguments are needed to rescue the casual hypothesis ". His style is well illustrated by the discussion of inhalation, which is given pride of place in the summary of the evidence. Among heavy smokers, inhalers suffer lung-cancer rates similar to or even, according to some studies, lower than non-inhalers. Professor Burch asserts that this alone refutes the hypothesis, but fails to mention alternative explanations such as differential distribution of particles between the bronchi, where most cancers originate, and the alveoli.

To demonstrate that all " paradoxes " are consistent with the conventional view is impossible in a brief review, but the competing models can be compared by examining the ratio of male to female lung cancer rates in, say, the 50–54 age-group in successive years. If the hundred-fold increase in reported rates merely reflects diagnostic improvement this ratio should have remained roughly constant, assuming that diagnostic standards are similar in men and women. If smoking causes cancer there should have been a progressive increase until about 1950, when male smoking rates had been fairly constant for 30 years and female rates had risen abruptly to about half the male rate; the ratio should subsequently fall to about 2:1 by 1980, when the young women who started smoking in 1950 reach the age of 50. The actual figures are: 1·7 (1913), 2·5 (1923), 4·7 (1933), 6·4 (1943), 8·9 (1953), 5·6 (1963), 3·3 (1973). I eagerly await Professor Burch's unconvincing *ad hoc* argument.

The main part of the remainder, comprising about a third of the book, is devoted to fitting a five-parameter model based on the idea that all cancers and many other diseases are the result of mutations in a growth control centre. By varying the parameters the predicted incidence can be made to increase progressively, flatten off or fall in the oldest age-groups, so the age-specific incidence rates of many diseases can be approximated, although the fit is often poor in the lowest age-groups. Most of the fitted curves turn down in old age in apparent confirmation of the underlying idea that a genetically susceptible subgroup is progressively eliminated, but in many cases this entails enthusiastic extrapolation up to 15 years beyond the observed rates, which rise steadily with increasing age. These analyses are irrelevant to the central growth control thesis, as the incidence rates could be equally well fitted by the simple power law corresponding to successive local pre-malignant cellular changes modified by elimination of susceptibles. The suggestion that inflexions in age-specific incidence rates reflect variations in genetic susceptibility is attractive, but this issue is obscured by the Procrustean model. The consequences of the simplistic assumption that individuals are susceptible or immune and the dubious inference that a mode in old age is genuine, and necessarily implies genetic susceptibility, are not discussed. Professor Burch is presumably not aware that the apparently minor modification of allowing the incidence in the low-risk group to increase from zero to 5% of the incidence in susceptibles virtually restores the log-linearity of the simple power law, while lower values can produce the intriguing inflexion of stomach cancer incidence in Japan which he ascribes to separate diseases. The theoretical as well as statistical robustness of fitting the power law is thus often well founded, irrespective of genetic effects, which are quite properly examined separately, so his contempt for the " widespread failure to appreciate that genetic inheritance cannot be disregarded " is rather misplaced. His own failure to appreciate the naivety of his model or the robustness of the analyses he dismisses is one of many " rather elementary lapses in scientific logic ", a phrase he applies to the belief that smoking causes lung cancer.

J. Peto